# Evaluating the Success of Preformed Resin Bonded Composite Strip Crowns for the Primary Central Incisor in Restoring Carious Primary Canine Teeth: A Retrospective Analysis

**DOI:** 10.7759/cureus.67012

**Published:** 2024-08-16

**Authors:** Balaji Suresh, Rajasekar Gunasekaran

**Affiliations:** 1 Pediatric and Preventive Dentistry, Saveetha Dental College and Hospitals, Saveetha Institute of Medical and Technical Sciences, Saveetha University, Chennai, IND

**Keywords:** early childhood caries (ecc), fdi criteria, primary canine, composite resin, strip crown technique

## Abstract

Background

Restoration of primary canine teeth in pediatric dentistry requires a balance of functional, biological, and esthetic factors. Stainless steel crowns, while effective for posterior restorations, often have patient acceptance issues due to esthetic limitations. Resin-bonded composite strip crowns have gained traction for anterior restorations due to their superior esthetic qualities and repairability. However, their long-term performance in primary canines characterized by unique morphological and functional demands has not been thoroughly evaluated. This study investigates the efficacy of resin-bonded incisor strip crowns in primary canines over three years, assessing their functional durability, biological integrity, and esthetic performance to provide a comprehensive evaluation of their long-term success in this application.

Methods

This retrospective observational study, conducted from September 2023 to December 2023 at the Department of Pediatric and Preventive Dentistry, Saveetha Dental College and Hospitals, Chennai, Tamil Nadu, India, received ethical approval from the Institutional Human Ethical Committee (IHEC/SDC/PEDO-2103/23/131). Dental records from January 2020 to December 2020 were reviewed by two investigators to identify children aged six years or younger who had undergone pulpectomy in primary canine teeth and were restored with resin-bonded incisor strip crowns. Exclusions included records with missing contact details or post-treatment radiographs. The purpose was to ensure a three-year review period when contacting patients in 2023. Eligible parents were contacted by phone, informed about the study, and invited for a review. Informed consent was obtained in person. Children with exfoliated primary canines and those whose parents did not sign the consent form were excluded. The restored crowns were evaluated using World Dental Federation (FDI) criteria across functional, biological, and esthetic domains by two calibrated examiners. Statistical analysis was conducted using IBM SPSS Statistics for Windows, Version 24 (Released 2016; IBM Corp., Armonk, New York, United States), with categorical variables expressed as n (%), inter-examiner reliability assessed by Cohen’s kappa, and chi-square test used for goodness of fit, setting significance at 95%.

Results

Of the 92 primary canines restored with resin-bonded incisor strip crowns, 60 children were evaluated. The retention rate at the three-year follow-up was 50 (83.3%). Functional evaluation revealed that 48 (80%) of crowns maintained surface texture, 42 (70%) preserved form and contour, and 52 (86.6%) demonstrated acceptable occlusion and wear. Biologically, 55 (91.7%) of the crowns had no secondary caries, and 56 (93.4%) had no dental hard tissue defects at the margin. Esthetically, 48 (80%) of crowns retained surface texture, 46 (76.6%) had a satisfactory color match, and 45 (71.6%) exhibited minimal marginal staining. Statistical analysis confirmed significant results for all functional and esthetic parameters (p < 0.05).

Conclusion

The technique of placing incisor strip crowns in the place of primary canines can be recommended considering its maintenance of functional, biological, and esthetic properties.

## Introduction

A successful restoration is not just about the maintenance of physiological, biological, and mechanical properties, but also includes the maintenance of esthetic properties [1]. Esthetic dimensions of a restoration play a significant role in a patient’s perception of the success of a restorative treatment [2]. Stainless steel crowns are being used for restoring posterior teeth after pulpectomy; however, they are considered to have poor patient acceptance due to their esthetic limitations [3]. Also, they are not generally considered for restoring anterior teeth as they affect the esthetic appearance of the teeth. Of late, resin-bonded composite strip crowns are being extensively used in pediatric dentistry for restoring anterior teeth [4,5].

These crowns are the first choice of crowns for pediatric dentists because of their superior esthetics and ease of performing repair works on chipped or fractured surfaces of the crown. On the other hand, few clinicians consider strip crowns as a technique-sensitive procedure due to the possibility of interferences of saliva and blood with the bonding of the crown [6]. Few retrospective studies were conducted to assess the longevity of resin-bonded composite strip crowns. One study concluded that about 80% of resin-bonded strip crowns lasted up to two years without any chances of de-cementation or fracture [7]. Another study proved that resin-bonded strip crowns had a retention rate of 88% at the end of 18 months [8]. Clinical trials also produced similar results and recommended the use of strip crowns in anterior teeth for better results [9,10].

However, these strip crowns are predominantly used only in incisors and restoration of primary canine teeth usually is done using zirconia crowns [11,12]. In clinical practice, pediatric dentists prefer zirconia crowns over resin-bonded strip crowns for restoring primary canines [13]. This could be probably due to their doubts about the durability, longevity, and reliability of resin crowns over primary canine teeth. Primary canines which have more shedding duration than primary incisors need to be covered with a crown for more years [14]. However, not many studies have evaluated the longevity of resin-bonded incisor strip crowns for more than a period of two years. Adding to this, the morphological variations of primary canines can also affect the retention of resin-bonded strip crowns. Despite these facts, resin-bonded incisor strip crowns are invariably being used at the place of primary canines in many clinics to enhance retention. However, their retention rate or other functional properties have not yet been scientifically evaluated. Such a technique of using incisor strip crowns in the place of primary canines needs to be substantiated with research evidence before recommending to all clinicians. Hence, a retrospective study was initially formulated to find out the prevalence of the use of resin-bonded incisor strip crowns in primary canines and that was followed by evaluating the functional, biological, and esthetic properties of those crowns.

## Materials and methods

Study design and ethical approval

This retrospective observational study was conducted between September 2023 and December 2023 at the Department of Pediatric and Preventive Dentistry, Saveetha Dental College and Hospitals, Chennai, Tamil Nadu, India. The study received ethical approval from the Institutional Human Ethical Committee (IHEC), with reference number IHEC/SDC/PEDO-2103/23/131.

Procuring dental records and reaching out to participants

Dental records from January 2020 to December 2020 were reviewed by two investigators using the computer database available in the Medical Record Room. Only records from the Department of Pediatric and Preventive Dentistry were included. The records were filtered to include children aged six years or younger who had undergone pulpectomy in primary canine teeth. Only those records showing restoration with resin-bonded incisor strip crowns were selected. The strip crowns (3M ESPE, St. Paul, USA) were filled using a pedo shade packable composite resin (Z100, 3M ESPE, St. Paul, USA). Records with missing contact details or without post-treatment radiographs were excluded. The purpose of the study was to ensure that a three-year review period was feasible when contacting patients in 2023. The parents of eligible children were contacted by phone, and the study’s purpose was explained. Parents who agreed to participate were invited to the department for a review of the restored primary canines. Informed consent was obtained from parents in person. Children whose primary canine teeth had been exfoliated and those whose parents did not sign the consent form were excluded from the study.

Evaluation of restored crowns using World Dental Federation (FDI) criteria 

A complete clinical examination was made for all children who appeared for review. The functional, biological, and esthetic properties of the strip crowns placed in primary canines were evaluated using FDI criteria [15] by a set of two examiners. The three domains (functional, biological, and esthetic) had five sub-domains for functional factors (retention, marginal adaptation, proximal contact point, form and contour, occlusion, and wear), three sub-domains for biological factors (presence of secondary caries, dental hard tissue defects at margin and hypersensitivity), and three sub-domains for esthetics (surface texture, marginal staining, and color match). All sub-domains were categorized based on a five-point Likert scale as clinically excellent/clinically good/clinically satisfactory/clinically unsatisfactory/poor. 

Training and calibration

The examiners were provided with proper theoretical and practical training by an experienced pediatric dentist for evaluating the restored teeth through FDI criteria. After training, they were calibrated as well. This ensured that all examiners had a similar reproducibility rate for the evaluation of restoration based on FDI criteria. 

Statistical analyses

The data obtained was entered in Microsoft Excel Spreadsheet and was subjected to statistical analysis using IBM SPSS Statistics for Windows, Version 24 (Released 2016; IBM Corp., Armonk, New York, United States). Categorical variables were expressed as n (%). Cohen’s kappa statistics were used to assess inter-examiner reliability. The chi-square test was used as a goodness of fit test. The level of significance was set as 95%.

## Results

About 456 case sheets meeting the inclusion criteria were retrieved. Of these, 364 (79.83%) of the primary canines were restored with zirconia crowns, and 92 (20.17%) were restored with resin-bonded incisor strip crowns. However, among the 92, only 60 parents gave consent to participate in the study, resulting in the inclusion of 60 children with resin-bonded incisor strip crowns on their primary canines. Among these children, 32 (53.3%) were boys, and 28 (46.6%) were girls. The age distribution of the children was as follows: 23 (38.3%) were six years old, 31 (51.6%) were seven years old, and 6 (10%) were eight years old.

Table 1 shows socio-demographic details of children with resin-bonded incisor strip crowns and zirconia crowns in primary canines.

**Table 1:** Socio-demographic details of children with resin-bonded composite strip crowns and zirconia crowns in primary canines.

		Resin-bonded incisor strip crowns placed in primary canines n (%)
Age	6 years	23 (38.3)
	7 years	31 (51.6)
	8 years	6 (10)
Gender	Boys	32 (53.33)
	Girls	28 (46.66)

Cohen's kappa was utilized to ascertain the reliability in assessing the quality of obturation between two examiners, showing a high level of reliability, κ = 0.82 (95% CI), p = 0.05.

Table 2 shows an inter-examiner reliability assessment using Cohen’s kappa statistics.

**Table 2:** Inter-examiner reliability assessment using Cohen’s kappa statistics. *p<0.05 - statistically significant

Reliability type	Method/statistic	κ value	p-value
Inter-examiner reliability	Cohen’s kappa coefficient	0.82	0.05

The evaluation of the functional properties of resin-bonded incisor strip crowns placed in primary canines demonstrated several significant findings. The retention rate was high, with 50 (83.33%) of the crowns rated as clinically good or satisfactory (p = 0.04). Marginal adaptation was observed to be favorable in 48 (80%) of the cases (p = 0.03), while 42 (70%) of the crowns exhibited acceptable proximal contact points (p = 0.01). Additionally, 36 (60%) of the crowns maintained proper form and contour (p = 0.02), and 52 (86.6%) demonstrated satisfactory occlusion and wear (p = 0.01). These results indicate that resin-bonded incisor strip crowns effectively maintain their functional integrity in primary canines over a significant period.

Table 3 shows the evaluation of functional domains of resin-bonded composite strip crowns during the third-year follow-up.

**Table 3:** Evaluation of functional domains of resin-bonded composite strip crowns during the third-year follow-up. Chi-square test; *p-value<0.05: statistically significant

Sub-domains	Resin-bonded incisor strip crowns placed in primary canines clinically good/satisfactory		p-value
	Yes	No	
Retention	50 (83.33)	10 (16.67)	0.04*
Marginal adaptation	48 (80)	12 (20)	0.03*
Proximal contact point	42 (70)	18 (30)	0.01*
Form and contour	36(60)	24 (40)	0.02*
Occlusion and wear	52 (86.6)	8 (13.4)	0.01*

The evaluation of the biological properties of resin-bonded incisor strip crowns placed in primary canines revealed promising outcomes. The presence of secondary caries was minimal, with 55 (91.7%) of the crowns rated as clinically good or satisfactory in this regard (p = 0.03). Furthermore, dental hard tissue defects at the margin were also minimal, with 56 (93.4%) of the crowns showing no significant defects (p = 0.02). These results suggest that resin-bonded incisor strip crowns exhibit strong biological compatibility and effectively protect against secondary caries and marginal defects.

Table 4 shows the evaluation of biological domains of resin-bonded composite strip crowns during the third-year follow-up.

**Table 4:** Evaluation of biological domains of resin-bonded composite strip crowns during the third-year follow-up. Chi-square test; *p-value<0.05: statistically significant

Sub-domains	Resin-bonded incisor strip crowns placed in primary canines clinically good/satisfactory		p-value
	Yes	No	
Presence of secondary caries	5 (8.3)	55 (91.7)	0.03*
Dental hard tissue defects at the margin	4 (6.6)	56 (93.4)	0.02*

The assessment of the esthetic properties of resin-bonded incisor strip crowns placed in primary canines demonstrated favorable outcomes. The surface texture was maintained at a high level, with 48 (80%) of the crowns rated as clinically good or satisfactory (p < 0.001). Marginal staining was minimal, with 45 (71.6%) of the crowns showing no significant staining (p < 0.001). Additionally, the color match of the crowns was well-preserved, with 46 (76.6%) of the crowns exhibiting a good or satisfactory color match (p < 0.001). These findings indicate that resin-bonded incisor strip crowns maintain their esthetic qualities effectively over time, providing a visually appealing restoration for primary canines.

Table 5 shows the evaluation of esthetic domains of resin-bonded composite strip crowns during the third-year follow-up.

**Table 5:** Evaluation of esthetic domains of resin-bonded composite strip crowns during the third-year follow-up. Chi-square test; *p-value<0.05 – statistically significant

Sub-domains	Resin-bonded incisor strip crowns placed in primary canines clinically good/satisfactory		p-value
	Yes	No	
Surface texture	48 (80)	12 (20)	<0.001*
Marginal staining	45 (71.6)	15 (18.4)	<0.001*
Color match	46 (76.6)	14 (23.4)	<0.001*

**Figure 1:**
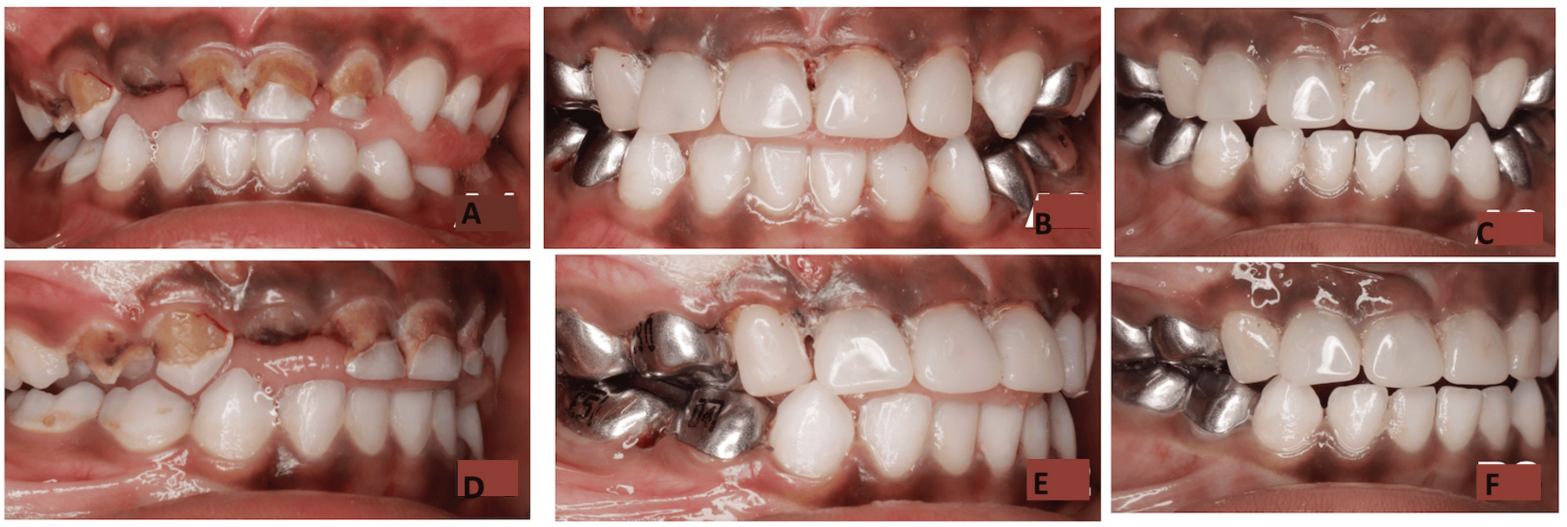
Clinical images of resin-bonded incisor strip crowns in primary canines. Preoperative images: frontal view (A) and right molar view (D); Immediate postoperative images: frontal view (B) and right molar view (E); Three-year follow-up images: frontal view (C) and right view molar (F). Note the shape of the canine with the incisor crown, demonstrating good gingival health and excellent marginal adaptation.

Figure 1 represents intraoral images of one of the patients assessed with the complete three-year follow-up.

## Discussion

This study evaluated the functional, biological, and esthetic properties of resin-bonded incisor strip crowns cemented in primary canine teeth. From the results, it is evident that retention and other functional properties of incisor strip crowns were maintained for up to three years with only a minimal number of crowns exhibiting secondary caries. It was also good to note that the color match and surface texture of the crowns lasted up to three years indicating their enhanced esthetic property. 

The study results were found to be in line with the study conducted by Ram et al., where an 88% success rate was proved for incisor strip crowns at the 18th-month follow-up [7]. Kupietzky in his study also assessed the longevity of incisor strip crowns and found a retention rate of 80% for incisor strip crowns at the two-year follow-up [5]. Grewal et al. also concluded that incisor strip crowns had a retention rate of over 80% at one-year follow-up [16]. However, all these incisor strip crowns were placed only in incisors and the present study is one of the first of its kind to assess the retention rate of incisor strip crowns placed in primary canines. Also, the incisor strip crowns were assessed at least after three years of cementation in the present study to ensure a longer follow-up is achieved. The time frame for record procurement was set in such a way that both three-year follow-up is ensured and the chances of exfoliation are also minimal.

Fracture of the material or crown is the most common reason for failure of a restoration [17]. It was speculated that incisor strip crowns designed exclusively for incisors may not bear masticatory forces in the canine area and may lead to fractures [18,19]. However, in the present study, most of the incisor strip crowns placed in canine teeth survived till three years without any fracture or extensive chipping. Also, other domains of functional properties such as marginal adaptation, proximal contact point, and form and contour were maintained by the incisor strip crowns. It is well known that resin-bonded strip crowns have good esthetics at the time of cementation, however, maintenance of appearance for one or two years is always questionable [20,21]. Surprisingly, most of the crowns showed good color match without any staining at the third-year follow-up. According to the study results, the incisor strip crowns had a very low rate of failures concerning biological domains such as the presence of secondary caries and dental defects. 

This study used FDI criteria to evaluate the strip crown’s success. However, failure of a crown/restoration is not just affected by retention and fracture but also due to other biological, functional, and esthetic properties. Hence, a comprehensive evaluation consisting of all dimensions was included. All examiners using FDI criteria were trained and calibrated to ensure the reliability of the results obtained. 

This study also has a few limitations. Firstly, this study was conducted as a retrospective study and hence the chances of bias and inaccuracy are higher. Studies with similar objectives need to be conducted prospectively to increase the accuracy and validity of the present findings. Clinical trials also must be conducted to prove the effectiveness of the use of resin-bonded incisor strip crowns in the place of primary canines. Secondly, the sample size was not determined for the present study as all the records that showed the use of resin-bonded incisor strip crowns in canine teeth were included. Thirdly, the absence of any comparison group can also be considered as a limitation. Future studies must be designed considering these limitations to obtain more accurate results. 

## Conclusions

This retrospective observational study evaluated the functional, biological, and esthetic properties of resin-bonded incisor strip crowns used to restore carious primary canine teeth over three years. The results indicated that these crowns exhibited a high retention rate, with minimal incidence of secondary caries and dental hard tissue defects. Additionally, the crowns maintained satisfactory surface texture, color match, and marginal staining, demonstrating good esthetic outcomes. Despite the retrospective design and absence of a comparison group, the findings suggest that resin-bonded incisor strip crowns can be a reliable and aesthetically pleasing option for restoring primary canines. 
